# Pectoralis Major Myocutaneous Flap for Head and Neck Defects in the Era of Free Flaps: Harvesting Technique and Indications

**DOI:** 10.1038/srep46256

**Published:** 2017-04-07

**Authors:** Muyuan Liu, Weiwei Liu, Xihong Yang, Haipeng Guo, Hanwei Peng

**Affiliations:** 1 Department of Head and Neck Surgery, Cancer Hospital of Shantou University Medical College, Shantou, 515031, P. R. China; 2 Department of Head and Neck Surgery, Cancer Center, Sun Yat-sen University, Guangzhou, 510080, P. R. China

## Abstract

The role of the pectoralis major myocutaneous flap (PMMF) in head and neck reconstruction is challenged recently due to its natural drawbacks and the popularity of free flaps. This study was designed to evaluate the indications and reliability of using a PMMF in the current free flap era based on a single center experience. The PMMF was harvested as a pedicle-skeletonized flap, with its skin paddle caudally and medially to the areola, including the third intercostal perforator, preserving the upper one third of the pectoralis major muscle. The harvested flap was passed via a submuscular tunnel over the clavicle. One hundred eighteen PMMFs were used in 114 patients, of which 76 were high-risk candidates for a free flap; 8 patients underwent total glossectomy, and 30 underwent salvage or emergency reconstruction. Major complications occurred in 4 patients and minor complications developed in 10. Tracheal extubation was possible in all cases, while oral intake was possible in all but 1 case. These techniques used in harvesting a PMMF significantly overcome its natural pitfalls. PMMFs can safely be used in head and neck cancer patients who need salvage reconstruction, who are high risk for free flaps, and who need large volume soft-tissue flaps.

Since its introduction by Ariyan in 1979, the pectoralis major myocutaneous flap (PMMF) has been used as a workhorse flap for the reconstruction of the head and neck defects in the following three decades^1^,^2^,^3^,^4^,^5^. Advantages of this flap include its easy harvest, abundant soft tissue volume, large skin paddle, relative versatility, considerable reliability, and short operating time. However, with the development of microvascular techniques and the wide use of free tissue transfers, drawbacks of the PMMF were magnified and its popularity in head and neck reconstruction decreased in the recent decades. Disadvantages of the PMMF include excessive bulk in some situations, deformity of the thoracic wall, function impairment of the neck and shoulder, high incidence of complications and partial necrosis of its skin paddle, and possibly poor function outcome of the recipient site^1^,^3^,^6^,^7^,^8^,^9^,^10^. The PMMF is now popularized in developing countries with limited medical resources^5^,^11^,^12^, whereas it is used much less in Western countries where availability of microsurgical techniques is more widespread^3^,^13^,^14^. It seems that the role of the PMMF in head and neck reconstruction has shifted from a “workhorse flap” to a “salvage flap” in the era of free flaps^9^.

However, we found in our practice that the role of the PMMF is irreplaceable, even though free flaps are our main armamentarium for the reconstruction of head and neck defects. It can safely be used not only as a “salvage flap” in cases with flap failure or complications (e.g. fistula and carotid rupture) but also as a primary procedure in patients who were predictably high risk candidates for a free flap, in situations where bulky flaps are needed (e.g. total glossectomy reconstruction), and in cases where simultaneous protection of the major vessels of the neck is necessary. Furthermore, technique modifications of flap harvesting not only ensure its reliability but also decrease the donor site functional impairment. Since January 2007, we have harvested PMMFs with a combination of previously reported modification techniques, and used the PMMF as not only as a salvage flap for failed free flap reconstructions, but also as a primary head and neck reconstruction flap when free flaps were thought to be a compromised choice. In the current cohort study, we report our experience of using 118 PMMFs in 114 patients by a single surgical team, focusing on flap harvesting techniques, indications, and surgical outcomes.

## Results

### Demographic data

From January 2007 through December 2015, 625 free flaps and 158 pedicled flaps (PMMF, n = 118; infrahyoid myocutaneous flap, n = 35; vertical trapezius myocutaneous flap, n = 5) were performed by a single surgical team (led by Dr.PH). These 118 consecutive PMMFs performed in 114 patients constituted our study subjects. Of the 114 cases, eighty were male and 34 were female. The age ranged from 45 to 94, with a median age of 64 years.

### Indications

Indications for a PMMF reconstruction were shown in Table 1. When there were multiple factors leading to the use of a PMMF, the principle one identified for the choice was listed as the indication. The majority of the PMMFs (73.8%, 87/118) were used as a primary reconstructive surgery, and the remaining (26.2%, 31/118) were used as a salvage or emergency procedure. Most of the primary PMMF reconstructions were chosen for those thought to be high-risk microvascular surgery candidates due to poor vascular status (41.4%, 36/87) and/or compromised general status (27.6%, 24/87). Fourteen PMMFs were used for coverage of the neck soft tissue defects or protection of the carotid artery following salvage extensive radical neck dissection in 12 patients. Five were used in patients with vessel depleted neck due to previous surgery. The remaining 8 flaps were employed for reconstruction for total glossectomy defects in 7 patients.

Salvage or emergency PMMF reconstruction was employed mainly for closure of the wound due to free flap failure (48.4%, 15/31), oro-/pharyngo-cutaneous fistula incurable after exhaustively conservative care (45.2%, 14/31), and for protection of the carotid artery in 2 cases of emergency carotid rupture.

In the 84 patients who underwent a primary PMMF reconstruction, 3 patients needed bilateral simultaneous PMMFs, 2 for carotid artery protection and 1 for total glossectomy defect. Clinical demographic breakdown of these 84 patients was detailed in Table 2. The surgical defects included oral cavity (38.1%, 32/84), hypopharynx (25%, 21/84), oropharynx (16.7%, 14/84), and head and neck soft tissue (20.2%, 17/84). The majority of patients had previous radiotherapy (57.1%, 48/84), recurrent malignancies (63.1%, 53/84), stage IV (84.5%, 71/84), and squamous cell carcinoma (95.2%, 80/84).

### Surgical outcomes

No perioperative deaths occurred in this series. Major complications developed in 2 patients with hematoma in the neck that needed surgical intervention, one with fistula that needed another PMMF from the contralateral side, and 1 with marginal necrosis that resulted in wound dehiscence and need for a delayed closure. Minor complications included marginal flap necrosis (n = 3), pharyngo-cutaneous fistula (n = 3), oro-cutaneous fistula (n = 3), and hematoma in the chest wall (n = 1), all of which were cured after conservative care. The major and minor complication rate were 3.5% (4/114) and 8.8% (10/114), respectively, with a total complication rate of 12.3%. All complications occurred in the primary reconstruction group. No total or partial flap necrosis and donor site dehiscence or infection occurred in our series. All patients resumed oral diet except 1 patient with total glossectomy combined with mandibulectomy and laryngectomy. Decannulation was possible in all patients except those who underwent total laryngectomy.

### Donor site morbidity

Donor site morbidity evaluation data of eighteen patients were unavailable due to death, tumor recurrence, poor general status, and unknown reasons. Ninety-six patients completed questionnaires in the outpatient department 6–12 months after operation. No patients reported severe shoulder pain or dysfunction in daily life. Moderate shoulder pain and moderate shoulder dysfunction were reported by 5 and 6 patients, respectively, among which 2 had both pain and dysfunction. With regard to cosmetic outcome of the donor site, 93.8% (90/96) of the patients reported as excellent or good.

## Discussion

The PMMF has been relegated to the secondary role in head and neck defect reconstruction in the recent two decades due to the widespread utilization of free flaps^1^,^6^,^15^. Free flaps are considered as the first choice in the majority of major head and neck defects because of their superior versatility, reliability, tissue match, function and cosmetic outcome, and lower donor site morbidity^16^. However, free flaps cannot be an all-in-one answer for head and neck reconstruction in any situation. Selection of an appropriate reconstructive method should take both patient factors and surgeon/institution factors into account. We believe when the reliability is assured and the donor site morbidity is decreased, the PMMF can be used not only as a workhorse flap for head and neck reconstruction in the institutes where availability of free tissue transfer is limited^7^,^17^,^18^, but also in select cases in institutes which preferentially use free flaps^3^,^13^,^19^.

In order to circumvent the natural drawbacks of the PMMF, technique modifications have been attempted^1^,^20^,^21^,^22^,^23^,^24^,^25^. We harvested the PMMF using the following techniques: the skin paddle is designed caudally-medially to the areola; the third intercostal perforating branch of the internal thoracic artery is included in the flap; the clavicle portion and part of the sternal portion of the pectoralis major is left intact; and the flap is sent to the recipient site via the sub-muscular tunnel over the clavicle and beneath the platysma flap after the clavipectoral fascia is divided. Although each of these modifications has been reported previously^1^,^20^,^21^,^22^,^23^,^24^,^25^, the combination of all these modifications results in the following advantages: (1) skin paddle caudally-medially to the nipple not only avoids using the potential compromised blood supply skin paddle over the rectus, but also reduces the cosmetic impairment at the donor site; (2) including the third intercostal perforating branch of the internal thoracic artery ensures the blood supply of the distal part of the skin paddle due to choke anastomosis of the internal thoracic artery and the thoracoacromial artery^23^; (3) in contrast to the previous reported modification of preserving only the clavicular portion of the pectoralis major muscle, we transect the pectoralis major horizontally along the muscular fiber axis at the level where the terminal of the pectoral branch could be identified and skeletonize the vascular pedicle to its origin. We have found this novel modification to have the benefit of further elongating the vascular pedicle length (the length of the pedicle reach 8–10 cm in our series), decreasing excessive bulk in the neck, and minimizing the deformity of and functional impairment of the donor site; (4) passing the flap via the sub-muscular tunnel over the clavicle avoids additional injury to the clavicle thus further reducing subsequent shoulder dysfunction. In this cohort of 118 PMMFs, no total or partial flap necrosis occurred, the total complication rate was 12.3% with a major complication rate of only 3.5%, and the patient reported donor-site-related morbidity was very low. These excellent outcomes demonstrate a considerable reliability and benefit of these techniques.

Bulky volume which is one of the drawbacks of the PMMF can serve as a priority in specific situations. PMMFs has been reported to be an appropriate option for reconstruction of extensive neck soft tissue defects resulting from ablative surgery for cervical metastasis involving the overlying skin, since it allows both large volume soft tissue coverage and protection of the carotid artery^26^,^27^. With regard to total or near total glossectomy, flaps with large tissue volume should be employed with the purpose of adequate tissue bulk restoration. These flaps include the anterolateral thigh flap^28^, deep inferior epigastric artery perforator flap^29^, PMMF^30^, and others. The PMMF is the most frequent donor site in our department. All 7 defects after total glossectomy with (n = 6) or without (n = 1) preservation of the larynx were successfully reconstructed using a PMMF; oral diet was possible in 6 patients except the patient who had a recurrent floor of the mouth cancer after chemoradiation and underwent total laryngectomy and bilateral mandibulectomy simultaneously.

In the institutions where defects are managed predominantly with free tissue transfer, the PMMF continues to play an important role in head and neck reconstruction. In his report of 53 PMMF reconstructions, Schneider *et al*.^3^ used the PMMF as secondary reconstruction for complications resulted from a free flap (e.g. flap necrosis or loss of soft tissue skin paddle, fistula, wound breakdown with great vessel exposure, and delayed hematoma), combined with a free flap for large tissue defects, and as primary reconstruction of cervical skin defect, great vessel coverage, pharyngo-cutaneous fistula, infection, and dead space obliteration. Avery *et al*.^13^,^14^ mainly used the PMMF in the management of advanced disease combined with substantial co-morbidity and situations following free flap failure. Regarding hypopharyngeal reconstruction for patients with primary or recurrent hypopharyngeal carcinoma after chemoradiotherapy, the PMMF has been reported to have benefits in terms of reliability and functional outcome, and less major complications^19^,^31^,^32^,^33^. In our institute, decision of a reconstructive method is based mainly on patient factors because both free tissue transfer and regional flap reconstruction (e.g. PMMF, infrahyoid myocutaneous flap, trapezius myocutaneous flap) are fully available^34^,^35^. Final decisions are made by a head and neck multidisciplinary board and accomplished by a single surgical team led by Dr.PH. During this cohort study period, 625 free flaps and 158 pedicled flaps (118 were PMMF) were performed for reconstruction of head and neck defects, the PMMF composed 15% of all reconstructions, slightly higher than most other institutions preferentially using free flaps^5^,^13^. Of all 118 PMMFs in our cohort, thirty-one were used as a salvage or emergency procedure with a purpose similar to other reports: closure of the wound due to free flap failure or refractory fistula and for protection of the carotid artery after an emergent rupture. Although use of a second free flap in management of a previous failed reconstruction has been reported to be reliable and effective with high success rate^36^, similar to most of the other institutions^3^,^5^,^9^,^13^,^37^, we preferred to manage this situation with a more reliable regional flap, avoiding possible vessel crisis due to the previously existing poor vascular status and the potential secondary infection of the surgical field. All the 29 cases with free flap complications were successfully salvaged by a PMMF in our series. Another indication of PMMF as an emergency salvage procedure is protection of the carotid artery following carotid rupture. Two cases with carotid rupture resulting from postoperative pharyngo-cutaneous fistula in our series were sent to the operating room for an emergent vascular repair and a PMMF was used to cover the fragile repaired artery. Both patients recovered uneventfully. We believe that the PMMF is a suitable donor site for this situation because of its easy harvest and ample muscular volume for major vessel protection even in an emergent situation.

The second category of indication for PMMFs is primary reconstruction for patients who are poor candidates for free flap reconstruction. They include: (1) patients with poor vascular status, e.g. poorly controlled diabetes, systemic vascular sclerosis, and/or previous high dose radiation to the neck; (2) patients with compromised general status due to old age or high ASA grade; (3) patients with vessel depleted neck due to previous surgery. Although studies on the impact of the aforementioned poor vascular status and compromised general status on free flap survival are controversial, a trend that these factors decrease successful flap outcome is apparent^38^,^39^,^40^,^41^. In addition, various techniques such as vascular selection and vein graft facilitate free flap reconstruction in vessel depleted neck^42^,^43^,^44^,^45^. However, all these techniques increase risk of a free flap failure. We prefer using a more reliable reconstructive method for these subjects rather than a potentially high-risk free flap, even though this may result in a less optimal functional and cosmetic outcome compared to a successful free flap. In the current series, 75% (65/87) PMMFs were chosen as a primary reconstruction mainly because of these factors.

## Conclusions

The aforementioned techniques in harvesting a PMMF result in minimization of its natural drawbacks, increased reliability and decreased donor site impairment. PMMFs can safely be used in head and neck cancer patients who need salvage or emergency reconstruction, who have high risk factors for a free flap, and select patients who need large volume tissue flaps such as after total glossectomy.

The flap harvesting techniques used in our series represent a combination of several previously reported modifications, with the novel addition of preservation of the sternocostal portion of the pectoralis major muscle. This is a retrospective cohort study without a control group, and the reliability and validity of donor site impair assessment is limited due to lack of initial study design. Further studies are needed to verify these conclusions.

## Methods

### Data collection and study approval

Clinical data were collected based on our institutional database. Demographic details, indications, pathological stage (AJCC TNM staging system, 2012), type of surgical resection, American Society of Anesthesiologists (ASA) grade, comorbidity, previous treatment, and flap complications were recorded.

This study was conducted with the approval of Medical Ethics Committee of Cancer Hospital of Shantou University Medical College, and was performed in accordance with the ethical standards of the Helsinki Declaration of 1975 and all subsequent revisions. Written informed consents were obtained from all patients before operation. All persons mentioned in the paper gave written informed consent for their data/images to be used for study and publication.

### Surgical techniques in harvesting a PMMF

**Figure 1:**
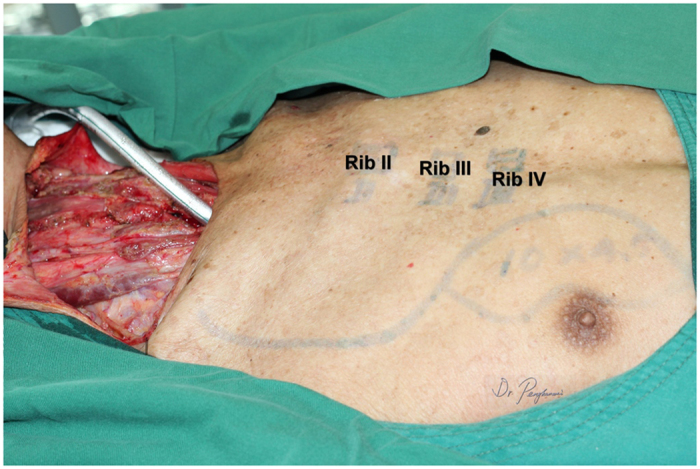
A partial circumferential defect of the hypopharynx resulted following ablative surgery for the recurrent hypopharyngeal squamous cell carcinoma after radical chemoradiation. The skin paddle was designed medially to the areola.

**Figure 2:**
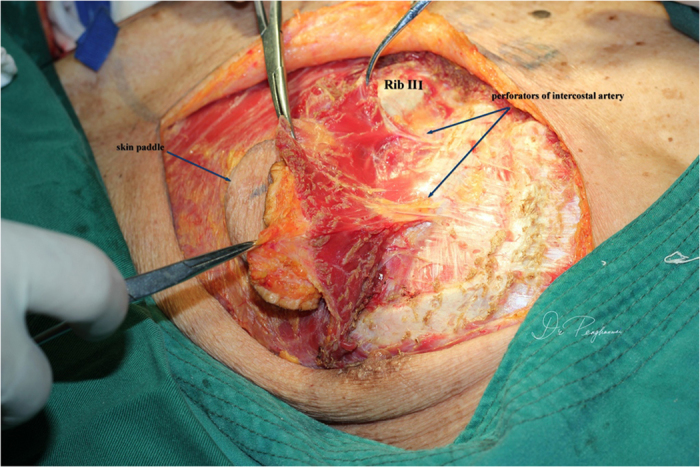
The third intercostal perforators from the internal thoracic artery was dissected and transected at its origins.

**Figure 3:**
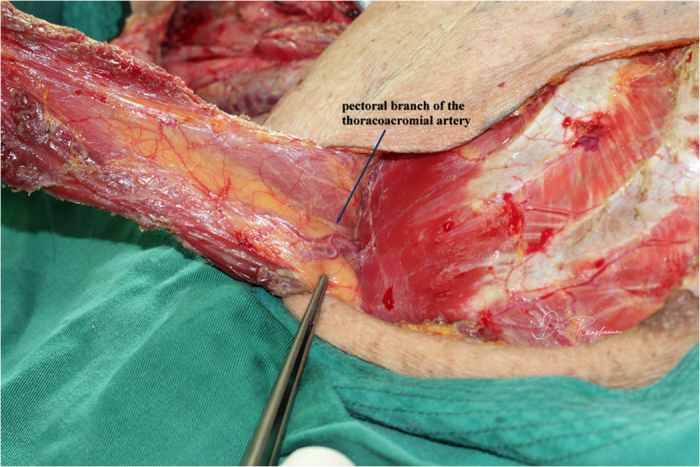
The pectoral branch of the thoracoacromial artery was identified beneath the pectoralis major.

**Figure 4:**
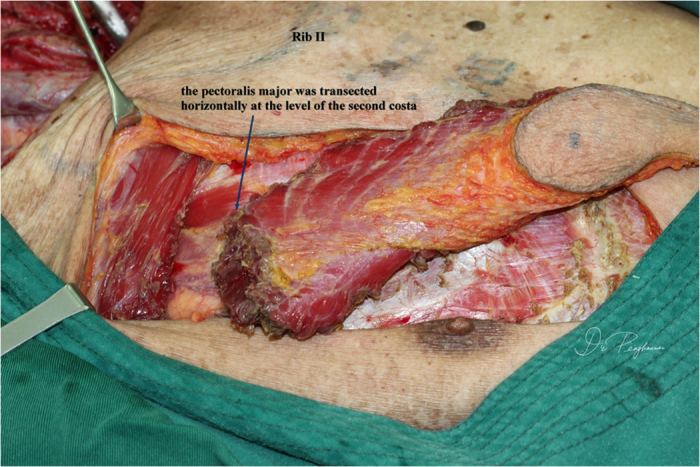
The pectoralis major muscle was transected at the level of the end of the pectoral branch and the pedicle was skeletonized, leaving its clavicle part and a portion of its sternocostal part intact.

**Figure 5:**
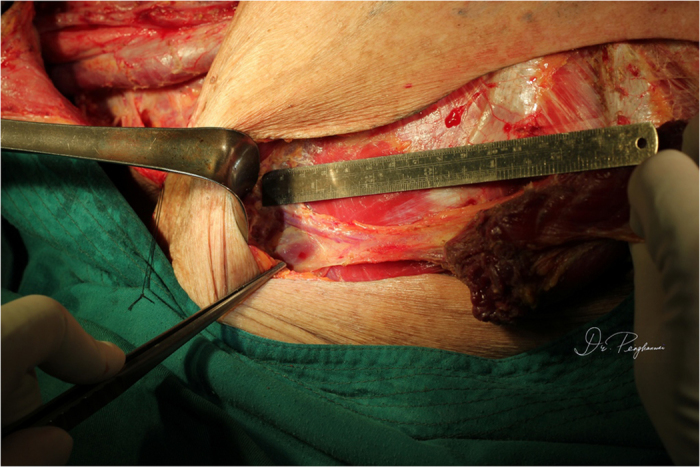
The length of the skeletonized pedicle was 10 cm.

**Figure 6:**
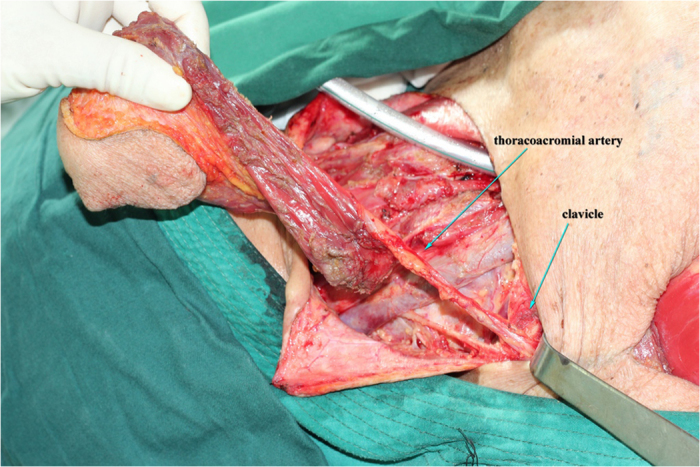
A tunnel was created over the clavicle beneath the pectoralis major muscle where the myocutaneous flap was passed through.

**Figure 7:**
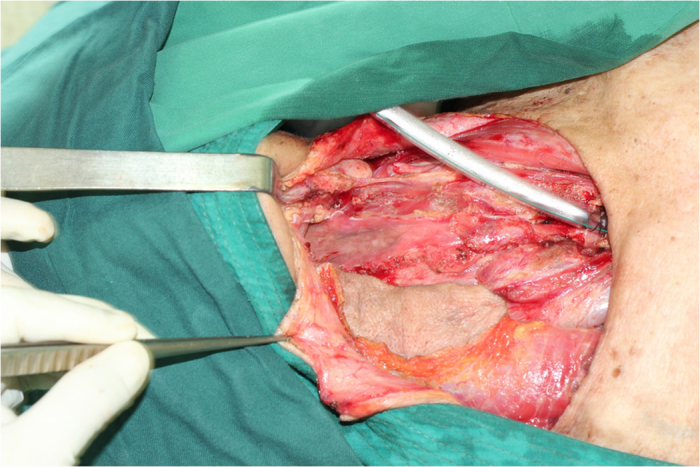
The PMMF was prepared at an appropriate position to repair the hypopharyngeal defect.

All patients underwent general anesthesia; tracheotomy was performed as indicated. The skin paddle was designed and marked over the chest wall caudally-medially to the nipple with sparing of the areola. The shape of the skin paddle matched the defect, mainly elliptically (Fig. 1). The inferior, medial, and lateral incision was made and the surrounding cutaneous flap was elevated to expose the pectoralis major. The attachment of the pectoralis major to the costa and the lower part of the sternum was detached and the space between the pectoralis major and minor was reached, keeping in mind that the third intercostal perforating branch of the internal thoracic artery was included and divided at the point where it derives from the chest wall (Fig. 2). Blunt dissection was performed to identify the pectoral branch of the thoracoacromial artery (Fig. 3). After extending the skin incision upward, the sternal attachment was divided. Then the pectoralis major was transected horizontally along the muscular fiber axis at the level where the terminal pectoral branch could be identified, commonly at the level of the second costa, leaving the upper one third (clavicle portion and part of the sternocostal portion) of the pectoralis major intact (Fig. 4). Vascular pedicle dissection was performed beneath the muscular fascia toward its origin, during which the external pectoral branches were sacrificed to skeletonize and elongate the vascular pedicle. The length of the pedicle reaches 8 to 10 cm (Fig. 5). After ligation of the perforator to the clavicular portion of the pectoralis major, the clavipectoral fascia was divided to create a tunnel. The flap was then passed to the defect region via the submuscular tunnel over the clavicle and beneath the platysma flap (Figs 6 and 7). The donor site defect can easily be closed in all the cases.

### Indications for a PMMF reconstruction

Indications for a PMMF rather than a free flap reconstruction as the first choice in our department in the study period from June 2007 to date included two categories: salvage or emergency procedure and primary reconstruction. Salvage PMMF reconstruction was considered in the following circumstances: (1) Failure of a free flap that needed another flap for closure of the wound; (2) Major complications such as fistula that was incurable without a surgical flap; (3) Protection of the Carotid artery in cases of carotid rupture for patients who underwent emergency vascular repair. Primary reconstruction with PMMF was considered when: (1) patients could not tolerate prolonged operation, mainly due to high ASA grade (3 and 4) and/or age older than 75 years with poor general status; (2) existence of potential high risk factors for free flap reconstruction, including long-term poorly controlled diabetes, systemic vascular sclerosis, and previous intensive radiotherapy (dose >60 Gy) to the neck; (3) patients with vessel depleted neck due to previous surgery; (4) coverage of the carotid artery to prevent accidental rupture in high risk patients, e.g. cervical metastasis with involvement of the overlying skin; (5) reconstruction for total glossectomy defects.

### Definition of complications

Surgical complications included major and minor complications. Major complications referred to total flap necrosis, partial necrosis, fistula, hematoma, incision dehiscence or infection that needed surgical intervention. Complications cured with conservative care were defined as minor complications.

### Donor-site-related morbidity

**Figure 8:**
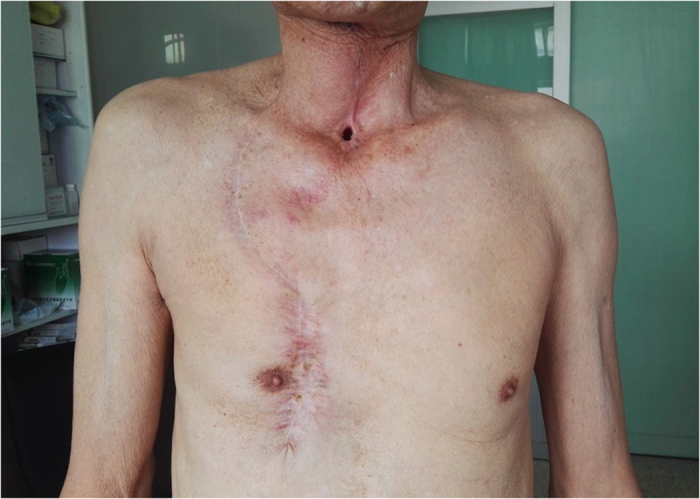
Six months after operation, the cosmetic and functional result was acceptable.

All patients were followed up every 3 months in the outpatient department. Donor-site-related morbidity was assessed and recorded by a trained outpatient nurse 6–12 months after surgery. We developed a 3-item questionnaire based on the Shoulder Pain and Disability Index^46^ and Lowery Scaling System^47^. To simplify the functional evaluation, the participants were asked to rate the severity of perceived daily shoulder pain and disability as none, mild, moderate, and severe; and the perceived chest wall cosmetic outcome was rated as excellent, good, fair, and poor (Fig. 8).

### Statistical analysis

Clinical data were input using SPSS version 16.0 software (SPSS, Inc., Chicago, IL, USA). Only descriptive statistics were employed. Numbers of patients and flaps were analyzed in appropriate situations respectively.

## Additional Information

**How to cite this article**: Liu, M. *et al*. Pectoralis Major Myocutaneous Flap for Head and Neck Defects in the Era of Free Flaps: Harvesting Technique and Indications. *Sci. Rep.* **7**, 46256; doi: 10.1038/srep46256 (2017).

**Publisher's note:** Springer Nature remains neutral with regard to jurisdictional claims in published maps and institutional affiliations.

## Figures and Tables

**Table 1:** Indications for a PMMF reconstruction.

Indications	Flap number	Percentage
Primary reconstruction	87	100%
Poor vascular status	36	41.4%
Previous radiation > 60 Gy to the neck	30	34.5%
Poorly controlled diabetes	4	4.6%
Systemic vascular sclerosis	2	2.3%
Compromised general status	24	27.6%
ASA grade 3–4	16	18.4%
Age > 75	8	9.2%
Major vessel protection	14	16.1%
Vessel depleted neck	5	5.7%
Total glossectomy	8	9.2%
Salvage reconstruction	31	100%
Flap failure	15	48.4%
Fistula	14	45.2%
Carotid rupture	2	6.5%

**Table 2:** Demographic data of 84 patients who underwent a primary PMMF reconstruction.

Types of defects	Number	Percentage
Oral cavity	32	38.1%
Mandibular	13	
Tongue (partial or total)	11	
Buccal mucosa	8	
Hypopharynx	21	25%
Oropharynx	14	16.7
Neck soft tissue	12	14.3%
Temporal region soft tissue	5	6.0%
Previous radiotherapy		
Yes	48	
No	36	
Primary tumor or recurrent tumor		
Primary	31	
Recurrent	53	
Tumor stage		
III	13	
IV	71	
Pathology		
SCC	80	
Non-SCC	4	
